# Label-free quantitative ^1^H NMR spectroscopy to study low-affinity ligand–protein interactions in solution: A contribution to the mechanism of polyphenol-mediated astringency

**DOI:** 10.1371/journal.pone.0184487

**Published:** 2017-09-08

**Authors:** Judith Delius, Oliver Frank, Thomas Hofmann

**Affiliations:** Chair of Food Chemistry and Molecular Sensory Science, Technical University of Munich, Lise-Meitner-Straße 34, Freising, Germany; George Washington University, UNITED STATES

## Abstract

Nuclear magnetic resonance (NMR) spectroscopy is well-established in assessing the binding affinity between low molecular weight ligands and proteins. However, conventional NMR-based binding assays are often limited to small proteins of high purity and may require elaborate isotopic labeling of one of the potential binding partners. As protein–polyphenol complexation is assumed to be a key event in polyphenol-mediated oral astringency, here we introduce a label-free, ligand-focused ^1^H NMR titration assay to estimate binding affinities and characterize soluble complex formation between proteins and low molecular weight polyphenols. The method makes use of the effects of NMR line broadening due to protein–ligand interactions and quantitation of the non-bound ligand at varying protein concentrations by quantitative ^1^H NMR spectroscopy (qHNMR) using electronic reference to access in vivo concentration (ERETIC 2). This technique is applied to assess the interaction kinetics of selected astringent tasting polyphenols and purified mucin, a major lubricating glycoprotein of human saliva, as well as human whole saliva. The protein affinity values (*BC*_*50*_) obtained are subsequently correlated with the intrinsic mouth-puckering, astringent oral sensation imparted by these compounds. The quantitative NMR method is further exploited to study the effect of carboxymethyl cellulose, a candidate “anti-astringent” protein binding antagonist, on the polyphenol–protein interaction. Consequently, the NMR approach presented here proves to be a versatile tool to study the interactions between proteins and low-affinity ligands in solution and may find promising applications in the discovery of bioactives.

## Introduction

**Fig 1:**
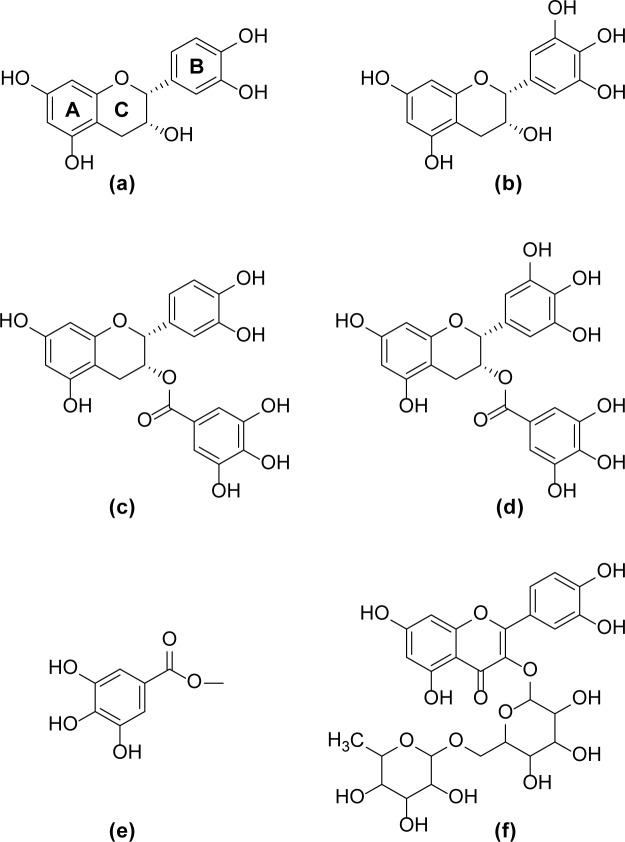
**Molecular structures of catechins and structurally related polyphenolic compounds.** (-)-epicatechin (a), (-)-epigallocatechin (b), (-)-epicatechin-3-gallate (c), (-)-epigallocatechin-3-gallate (d), methyl gallate (e), and quercetin-3-O-rutinoside (f).

Polyphenols account for the astringent sensation induced by plant-derived foods and beverages, such as persimmons [1], red currants [2], bananas [3], black tea [4], and red wine [5]. Typical examples of astringent phytochemicals are (-)-epicatechin (a), (-)-epigallocatechin (b), (-)-epicatechin-3-gallate (c), (-)-epigallocatechin-3-gallate (d), methyl gallate (e), and quercetin-3-O-rutinoside (f). The structures of these are shown in Fig 1.

According to the prevalent hypothesis of how oral astringency is mediated, polyphenols elicit astringency by interacting with salivary proteins, resulting in protein aggregation and precipitation [6]. Mediated by hydrogen bonding and hydrophobic forces between primarily aromatic regions of the polyphenol and proline-rich protein domains, polyphenols rapidly bind to salivary proteins and form aggregates, which can grow to colloidal sizes [7, 8]. However, the literature on the role of protein precipitation in the sensation from astringent molecules is rather contradictory. While some researchers reported a correlation between the astringency induced by polyphenols and their ability to precipitate salivary proteins [9, 10], others could not observe any conclusive relationship, indicating that protein precipitation may not be an essential event in oral astringency perception [11–13].

As the formation of soluble complexes between astringent polyphenols and salivary proteins is hypothesized as a candidate key event inducing oral astringency [14], the present study aims at characterizing the formation of soluble polyphenol–protein complexes. Nuclear magnetic resonance (NMR) spectroscopy is used here to investigate the interaction of polyphenols with salivary proteins in solution, since this spectroscopic technique is well-suited to characterizing weak affinity ligand binding, covering interactions with protein–ligand dissociation equilibrium constants (K_D_) in the micro- to millimolar range [15]. Previous NMR-based studies on polyphenol–protein interactions provided pioneering insight into the main binding sites using intermolecular nuclear Overhauser effects [16, 17] and chemical shift changes observed for small synthetic peptides upon ligand binding [17]. However, the model systems used were limited to purified, small oligopeptides [17–19], which do not necessarily reflect the behavior of native salivary constituents.

As conventional NMR-based binding assays may require cost-intensive isotopic labeling of the potential binding partners [20], we here introduce a label-free, ligand-focused quantitative NMR (qNMR) titration method to trace soluble complexes and estimate binding affinities between selected low molecular weight polyphenols and purified mucin, a major lubricating glycoprotein of human saliva, as well as the whole human salivary proteome. The method is based on monitoring the ^1^H NMR signals of a fixed amount of unlabeled ligand in the presence of an increasing concentration of native proteins. As the ^1^H NMR signals of the low molecular weight ligand vanish upon protein binding [21], integrating the remaining ligand signals provides a measure of the amount of unbound ligand for each titration step and, after fitting the binding isotherms, enables calculation of the affinity (*BC*_*50*_) of low-affinity ligands such as polyphenols for the protein. Besides recording data points for entire binding isotherms, the quantitative NMR method was tested for its suitability to assess the relative binding strengths of polyphenols with human whole saliva by single-point NMR measurements. Finally, a ^1^H NMR competitive binding assay was performed to study the known astringency-masking effect of carboxymethyl cellulose and to test this qHNMR approach as a versatile method for protein–polyphenol complexation studies.

## Materials and methods

### Chemicals

The following chemicals were commercially obtained: KH_2_PO_4_ (Merck KGaA, Darmstadt, Germany); Na_2_HPO_4_, citric acid, NaN_3_, sodium carboxymethyl cellulose (CMC, molecular weight ≈ 250 kDa), mucin from bovine submaxillary glands (type I-S) with a molecular weight between 0.4 and 4.0 MDa [22, 23], (-)-epicatechin (EC), (-)-epigallocatechin-3-gallate (EGCG), methyl gallate, saccharin (Sigma-Aldrich, Steinheim, Germany); quercetin-3-O-rutinoside (rutin) (Carl Roth, Karlsruhe, Germany); (-)-epicatechin-3-gallate (ECG), (-)-epigallocatechin (EGC) (Extrasynthese, Genay, France); acetonitrile (J.T. Baker, Deventer, Netherlands); D_2_O, DMSO-d_6_, and 3-(trimethylsilyl)propionic acid sodium salt (TMSP) (Euriso-top, Gif-sur-Yvette, France). All chemicals were of analytical grade or better and were used without further purification. Ultrapure water was prepared with a Milli-Q Gradient A10 system (Millipore, Schwalbach, Germany).

### Human saliva collection and preparation of salivary mucin solutions

Unstimulated whole saliva was freshly collected on ice from six healthy, non-smoking volunteers, who did not consume any food or beverages besides water for at least one hour prior to saliva sampling. The volunteers gave their informed written consent to participate in this study. Ethical approval was given by the ethics committee of the Technische Universität München (application #2311/09). Saliva samples were pooled and centrifuged twice (4,800 g for 5 min, then 16,000 g for 30 min, at 4°C) to remove cells and debris. The clear supernatant was adjusted to pH 7.0 by mixing in a 9:1 ratio (v/v) with a ten-fold concentrated buffer solution containing KH_2_PO_4_ (1.5 mol/L), NaN_3_ (2.0 mmol/L), and TMSP (5.8 mmol/L) in D_2_O. This buffered saliva, from now on referred to as saliva, was used for NMR binding studies. To calculate molar mucin concentrations, the arithmetic mean (2.2 MDa) of its reported molecular weight distribution [22, 23] was defined as the average molecular weight of mucin. A mucin solution (2.5 μmol/L) was prepared with a 1:10 (v/v) diluted potassium phosphate buffer (pH 7.0), filtered through a 0.45 μm polyether sulfone membrane (Pall, Crailsheim, Germany), and then diluted with phosphate buffer to adjust the protein content for the NMR titration series.

### Quantitative ^1^H NMR spectroscopy (qHNMR)

Aliquots (10 μL, 250 mmol/L) of the stock solutions of EC, EGCG, ECG, EGC, rutin, and saccharin in DMSO-d_6_ were mixed with buffered saliva (990 μL). Samples were incubated at room temperature for 15 min to allow the system to reach equilibrium. The concentration of unbound ligand was then determined by means of qHNMR using the NOESYPRESAT pulse sequence (*noesygppr1d*) for water suppression on a Bruker Avance III 500 MHz system (Bruker, Rheinstetten, Germany) equipped with a cryo-TCI probe (with z-gradient coils) at 300 K (controlled by a BCU 05) in precision NMR USC tubes (5 × 178 mm, Bruker, Faellanden, Switzerland) without rotation. Acquisition parameters have been set as follows: size of the fid = 64 k, spectral width = 19.9947 ppm, receiver gain = 90.5, acquisition time = 3.28 s, and relaxation delay = 20 s. Data were referenced to the ^1^H NMR signals of an equimolar ligand sample, which was prepared in protein-free phosphate buffer (control). Since the 90° pulse is inversely proportional to the ^1^H NMR signal intensity, the absolute concentration of the unbound ligand could be precisely calculated using the ERETIC 2 tool of the TopSpin 3.0 software (Bruker) based on the pulse length-based concentration determination (PULCON) methodology [24, 25]. High quality spectra were recorded by manually tuning and matching the NMR probe to a 50 Ω resistive impedance, which minimized the radio frequency (RF) reflection with the sample in place. T_1_ relaxations of all protons of interest were determined by using the inversion-recovery pulse sequence, acquiring 10 recovery delays between 0.01 and 15 s between the scans. The relaxation delay was set to five times the longest T_1_ relaxation time of the respective ligand to ensure complete proton relaxation. The following discrete NMR signals were not superimposed by protein resonances and were therefore selected for signal integration and ligand quantitation: EC: 6.11, 6.15, 7.00, and 7.07 ppm; ECG: 6.19, 6.90, and 7.03 ppm; EGC: 6.14, 6.18, and 6.69 ppm; EGCG: 6.20, 6.66, and 7.04 ppm; methyl gallate: 7.17 ppm; and rutin: 6.16, 6.88, and 7.43 ppm. For each compound, the integrals of the indicated aromatic signals were averaged.

### Ligand-focused qHNMR spectroscopic titrations

To record data for a complete binding isotherm, mucin was titrated from 0 to 2.5 μmol/L or until the free ligand concentration reached a plateau, whereas the ligand content (2.5 mmol/L) remained constant. The samples were freshly prepared in random order and measured at constant time intervals by qHNMR as described above.

### Quantitative analysis of NMR titration data

The cooperative drug-protein binding Hill model [26] was adopted to fit the experimentally observed data, describing the fraction of protein-bound ligand *θ*_*B*_, which is in dynamic equilibrium with the unbound ligand [*L*]_*free*_, as a function of the protein present [*P*]:

$$\theta_{B}=\frac{{\left[L\right]}_{total}-{\left[L\right]}_{free}}{{\left[L\right]}_{total}}=\frac{[{P]}^{\alpha }}{[P{]}^{\alpha }+{{(BC}_{50})}^{\alpha }}$$ (1)

*BC*_*50*_ corresponds to the protein concentration with half-maximal binding of the ligand and *α* is the Hill coefficient of sigmoidicity. If [*P*] equals *BC*_*50*_, 50% of the total amount of ligand [*L*]_*total*_ is bound. As salivary proteins such as mucins have a high molecular weight of up to several MDa [27], the total number of available protein binding sites is assumed to be greatly in excess compared to the concentration of the ligand [*L*]. Thus, the amount of unbound protein [*P*]_*free*_ is negligibly depleted by complexation and corresponds to the total amount of protein [*P*]_*total*_ present. The concentration of free ligand [*L*]_*free*_ is determined directly by the NMR experiment and the bound ligand mole fraction *θ*_*B*_ is calculated from this. Both *BC*_*50*_ and the shape parameter *α* were fitted with Gnuplot Version 5.0 (http://www.gnuplot.info), a public domain software for data visualization and curve fitting.

### Competitive qHNMR binding experiments

To study the effect of CMC on the interaction between EGCG and salivary proteins, aliquots (10 μL) of the EGCG stock solution were spiked to 1% (w/v) CMC in KH_2_PO_4_ buffer and 1% (w/v) CMC in buffer mixed with 10% saliva. As references, the same amount of EGCG stock solution was spiked to 990 μL of pure phosphate buffer and buffer containing 10% saliva. After equilibration for 15 min, qHNMR analysis was performed as described above.

### High-performance liquid chromatography (HPLC)

**Fig 2:**
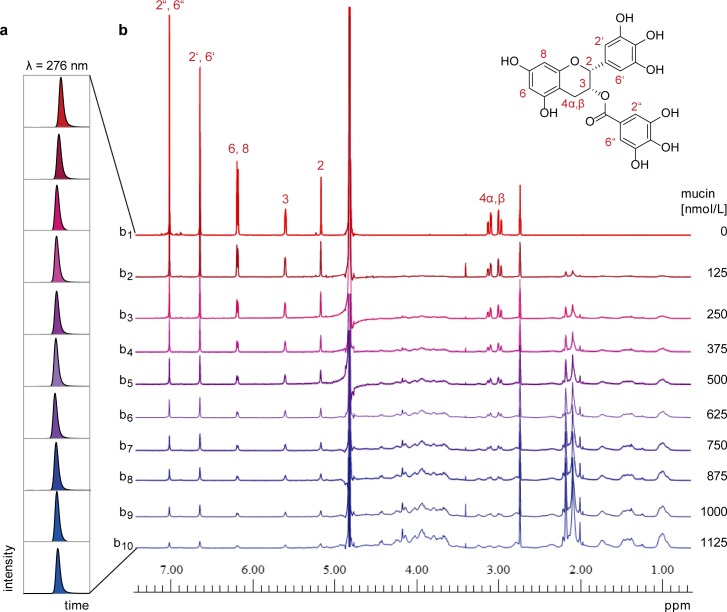
Analysis of (-)-epigallocatechin gallate (2.5 mmol/L) upon titration with salivary mucin. (a) Excerpts of the HPLC-UV chromatograms (λ = 276 nm) showing equal peak areas for EGCG that are independent of the protein concentration. (b) ^1^H NMR (NOESYPRESAT, H_2_O/Buffer (9:1, v/v), 300 K) spectra of EGCG in phosphate buffer (pH 7.0). The NMR signals of free EGCG in the absence of mucin are shown in spectrum b_1_. Sharp proton signals are clearly visible and are assigned to the molecular structure of EGCG being well in line with literature data [32]. As mucin is titrated to the sample, the proton signals of EGCG decrease (spectra b_2_−b_10_) until they merge with the baseline. NMR signals between 4 and 5.5 ppm are partially suppressed by the water suppression technique.

EGCG was additionally quantified using HPLC with UV detection (HPLC-UV). Aliquots (100 μL) of the samples prepared for NMR spectroscopy were diluted 1:5 (v/v) with citric acid-phosphate buffer (pH 4.0) and separated on a Jupiter RP-C18 column (250 × 4.6 mm; 5 μm; 300 Å; Phenomenex, Aschaffenburg, Germany) using a Jasco HPLC system (Jasco, Groß-Umstadt, Germany) with two Jasco PU-2087 pumps and a 7725i type Rheodyne injection valve (Rheodyne, Bensheim, Germany). Chromatograms were recorded with a Jasco DAD MD-2010 diode array detector (λ = 220−400 nm) at a wavelength of 276 nm (Fig 2A). Chromatography was conducted at a flow rate of 1.0 mL/min using acetonitrile (ACN) and water (both containing 0.1% formic acid) as a mobile phase, with a stepwise linear gradient from 5% to 20% ACN in 15 min to 100% ACN in 7 min.

### Sensory analysis

Sensory experiments were performed in a sensory laboratory at 20–22°C using a half-tongue test procedure [4, 28–30] by 12 trained sensory experts. The test solutions were freshly prepared at equimolar concentrations (5.0 mmol/L) in bottled water, which was also used for taste-neutral mouth rinsing. A higher polyphenol concentration was used than in the *in vitro* study to take into account the fact that the samples are diluted with saliva during oral administration. Aliquots (100 μL) of the test solutions were randomly administered on each half of the tongue. Panelists were asked to locate the more astringent solution on their tongue. Solutions of the astringents EC, ECG, EGC, EGCG, and methyl gallate were compared as such among each other, and rankings were assigned by adding up the scores of each compound.

## Results and discussion

Upon binding of a small-molecule ligand to a high molecular weight protein, the NMR signals of the ligand are known to become broad and the NMR signal integrals are attenuated as the protein concentration is incrementally increased. Integrating the residual NMR signals of the ligand in the presence of protein provides a measure of the amount of free ligand that is in dynamic equilibrium with the protein-bound state [21]. Initially, NMR titration experiments were performed to study the interaction of mucin, a major lubricating glycoprotein of human saliva [31], and EGCG, a key astringent compound of green tea leaves, in buffered solution (pH 7.0). In comparison, a sample lacking protein served as an external standard and accounted for the maximum concentration of free polyphenol. As expected, the ^1^H-NMR spectrum of purified EGCG revealed proton resonances as sharp signals (Fig 2B, spectrum b_1_).

While keeping the EGCG content constant, the protein concentration was subsequently varied for each NMR experiment. As shown in Fig 2B (spectra b_2_−b_10_), the ^1^H NMR signals of EGCG decreased with increasing mucin concentrations. For each titration step, the proportion of unbound EGCG was determined with a maximum technical error of < 2% [24] by integrating specific resonance signals, which were not superimposed by NMR signals of the protein, and comparing the peak areas with those of the corresponding reference sample. As polyphenol–protein interactions have been frequently studied by means of HPLC [13, 33, 34], samples prepared for NMR spectroscopy were additionally analyzed by HPLC-UV for a cross-method comparison. Using this orthogonal analytical technique, only one peak appeared in the HPLC chromatogram. The EGCG content was revealed to be identical in the mucin-free control and in all samples containing protein (Fig 2A), which reflects not the bound but the total polyphenol concentration in each sample. Apparently, polyphenol was released from the complex during chromatography due to the dynamic equilibrium between loosely associated complexation partners. As the free and protein-bound states of the polyphenol could not be differentiated, HPLC analysis may be considered inappropriate for monitoring such dynamic protein–polyphenol interactions. In comparison, the NMR-based approach allowed the absolute amount of unbound ligand to be determined, which in turn revealed its protein binding affinity.

### Structure-affinity relationships of polyphenol–protein binding

**Fig 3:**
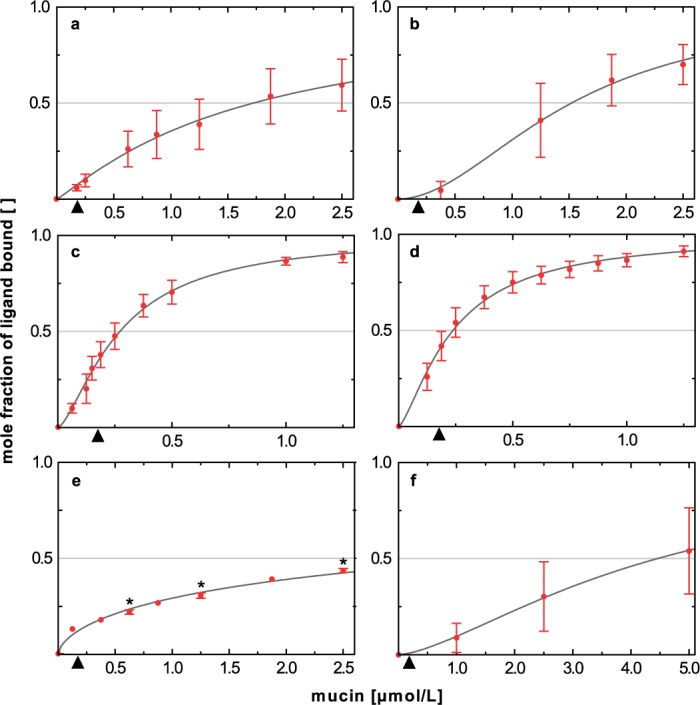
Fitted binding isotherms of polyphenols titrated with increasing levels of salivary mucin. The mole fraction of polyphenol bound to mucin is plotted against the protein concentration. The concentration of EC (a), EGC (b), ECG (c), EGCG (d), methyl gallate (e), and rutin (f) was kept constant (2.5 mmol/L), whereas salivary mucin was titrated from 0 to 2.5 μmol/L or until the bound mole fraction of the ligand reached a plateau. The approximate mucin content of human whole saliva is marked by an arrow. The intercept of the fitted binding isotherm with the horizontal gray line marks the half-maximum binding concentration of the protein (*BC*_*50*_). Error bars denote the standard deviation obtained from integrating all the distinct, non-overlaid proton signals of each compound (see methods for a list of consulted proton resonance signals). For methyl gallate only one discrete ^1^H resonance signal is available for integration. *Error bars in (e) denote the technical error of two independent measurements. Data points behind means are provided in S1 Table. Binding isotherms, the *BC*_*50*_ value, and the shape parameter *α* were fitted with the Gnuplot software (Version 5.0).

To systematically study the influence of the polyphenol structure on the binding affinity for salivary mucin and soluble aggregate formation, the ligand-focused NMR spectroscopic titration technique was applied to the structurally related polyphenolic compounds EC (a), EGC (b), ECG (c), EGCG (d), methyl gallate (e), and rutin (f) (Fig 1). The NMR spectra of all polyphenols tested were affected by the presence of mucin, and the molar fractions of bound polyphenol were determined as a function of the protein concentration (Fig 3).

Among the polyphenols, the galloylated flavan-3-ols ECG and EGCG showed the lowest *BC*_*50*_ values of 0.3 and 0.2 μmol/L, respectively, thus demonstrating by far the strongest binding affinity for salivary mucin (Table 1). This confirms that a galloyl moiety at position 3 of the flavan-3-ol scaffold contributes particularly to a high protein binding affinity [35], as this moiety enhances the structural flexibility of the polyphenol [36]. Both ECG (c) and EGCG (d) displayed similarly shaped, characteristic binding isotherms with a shift from low to high affinity with increasing mucin concentration. Compared with ECG and EGCG, a six times lower binding affinity was found for EGC (b), followed by EC (a), and finally, methyl gallate (e). The additional phenolic hydroxyl group located at the B-ring of EGC (*BC*_*50*_ ≈ 1.5 μmol/L) improved the interaction with the protein as compared to EC (*BC*_*50*_ ≈ 1.7 μmol/L). The NMR signals of rutin (f) were only weakly affected by mucin, and this displayed the lowest protein affinity (*BC*_*50*_ ≈ 4.5 μmol/L) compared with all the other astringents tested.

**Table 1:** Fit parameters of polyphenol–mucin binding isotherms.

Ligand [polyphenol]	*BC*_*50*_ [nmol/L]*	α*
(-)-epicatechin (EC)	1720 (± 60)	1.11 (± 0.06)
(-)-epigallocatechin (EGC)	1520 (± 50)	1.93 (± 0.15)
(-)-epicatechin gallate (ECG)	271 (± 7)	1.48 (± 0.06)
(-)-epigallocatechin gallate (EGCG)	238 (± 6)	1.40 (± 0.05)
methyl gallate	4200 (± 500)	0.63 (± 0.05)
rutin	4450 (± 80)	1.51 (± 0.06)

Fitted half-maximum binding constants (*BC*_*50*_) and shape parameters α describing the binding interaction of structurally related polyphenols with salivary mucin. The error of the fit parameters is indicated.

*Parameters were fitted with the Gnuplot software (Version 5.0).

### Affinity ranking by single-point qHNMR measurements

**Fig 4:**
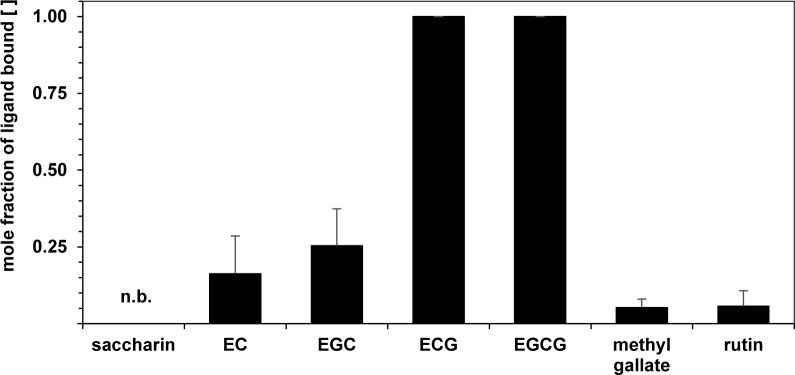
Mole fractions of polyphenols bound to human saliva proteins as assessed by single comparative qHNMR measurements. Aliquots (10 μL, 2.5 mmol/L) of the polyphenolic compounds EC, EGC, ECG, EGCG, methyl gallate, rutin, and saccharin were each mixed with pooled and centrifuged, non-stimulated saliva (990 μL). As crude saliva was previously blended with phosphate buffer in a 9:1 ratio (v/v), the total saliva content was 89.1%. Samples lacking saliva served as references. Error bars denote the standard deviation obtained from two independent measurements of biological replicates, integrating all distinct, non-overlaid proton signals of each ligand (see methods for a list of consulted proton resonance signals and S2 Table for the data points behind means). Saccharin showed no binding (n.b.) with human saliva.

As chemically unstable compounds such as polyphenols, as well as unstable biological material such as saliva, require fast sample handling to prevent degradation [37], single-point qHNMR analyses were tested for their suitability to assess the relative binding affinity of selected ligands with pooled human saliva. As the sweetener saccharin, which did not impart any astringent oral sensation, was expected not to interact with salivary proteins, this compound served as a negative control. The individual ligands were mixed with human saliva at equimolar concentrations, and the percentage of free ligand was determined for each sample in comparison to a reference sample prepared without saliva. The mole fractions of bound ligand were calculated with respect to the corresponding reference sample (Fig 4). The binding affinity of the ligands for human whole saliva follows the order EGCG ≈ ECG >> EGC ≈ EC > methyl gallate ≈ rutin, with no measurable interaction observed between human saliva and saccharin. Trends in binding strength deduced from the NMR titration experiments with mucin are thus largely reflected by the single-point NMR analysis with human whole saliva.

### Implications for the molecular mechanism of oral astringency

The results obtained with pooled human saliva are particularly relevant in terms of better understanding the mechanism of polyphenol-induced astringency perception. The catechins and methyl gallate were additionally sensorially characterized and ranked at equimolar concentrations according to their level of astringency as follows: EGCG ≈ ECG > EGC ≈ EC ≈ methyl gallate (Table 2). This essentially reflects the binding strength with human saliva determined by NMR spectroscopy. In accordance with qHNMR spectroscopic data obtained with human saliva, the astringent sensations induced by EGCG and ECG could not be differentiated by the sensory panel. Likewise, the trained test panel was not able to distinguish between the astringent orosensations imparted by EGC, EC, and methyl gallate. Furthermore, the ranking order reflects the recognition threshold concentrations reported for the orosensory astringency induced by EGCG (190 μmol/L) and the non-galloylated catechins EGC and EC (≈ 470 μmol/L) [38], thus confirming the particular role of the gallate moiety in the astringency of flavan-3-ols.

**Table 2:** Pairwise comparison matrix to assess the relative astringency of polyphenols.

as compared to	EC	EGC	ECG	EGCG	methyl gallate	score
**EC**		0	−1	−1	0	−2
**EGC**	0		−1	−1	0	−2
**ECG**	1	1		0	1	3
**EGCG**	1	1	0		1	3
**methyl gallate**	0	0	−1	−1		−2

An entry “1” indicates the polyphenol in the row to be more astringent than the polyphenol of the corresponding column at an equimolar concentration (5.0 mmol/L), “−1” indicates the polyphenol in the row to be less astringent as compared to the polyphenol in the respective column, and “0” implies no difference between the pairs. Polyphenols were overall ranked in astringency by horizontally adding up the scores of each compound: EGCG (3) ≈ ECG (3) > EGC (−2) ≈ EC (−2) ≈ methyl gallate (−2).

Comparing the mole fraction of the polyphenol bound to saliva (Fig 4) with the fraction bound at a mucin concentration of 0.4 mg/mL (Fig 3), which corresponds approximately to the mucin content in human whole saliva [39], revealed a relatively higher binding capacity for saliva, in particular for polyphenols bearing a gallate moiety. This may indicate that apart from mucin [40], other salivary proteins interacted with these polyphenols and contributed to the observed NMR signal attenuation in the presence of saliva. Accordingly, in addition to mucins, which account for about 16% of the total salivary protein content [39], particularly amylase [41] and proline-rich proteins [35], besides several other salivary proteins, have been recently shown to interact, for instance, with EGCG [42]. The molar fractions of methyl gallate and EC bound by 0.4 mg/mL mucin approximated the respective percentage bound by saliva, indicating that for these compounds, salivary mucin may contribute notably to the overall binding capacity of saliva. For rutin-mucin mixtures, soluble complexes were detected only at an artificially high mucin concentration. Flavonol glycosides elicit a velvet-like, mouth-coating astringent sensation, which stands out from the mouth-puckering astringent impression described for catechins [4, 43]. This may indicate that flavonol glycosides mediate a velvety type of astringency that is not caused by interacting with lubricating mucins. More likely, other salivary proteins are involved in the molecular mechanism of velvety astringency. Based on qHNMR spectroscopy, the formation of exclusively soluble polyphenol salivary protein complexes can be reasonably assumed for the polyphenols EC, EGC, methyl gallate, and rutin, which did not develop any centrifugable haze with saliva. Therefore, protein precipitation is probably not a prerequisite for the development of polyphenol-induced astringency.

### Competitive binding experiments

Polysaccharides, such as CMC, are known to reduce the mouth-puckering sensation elicited by astringent alimentary products [44–46]. Based on a combination of fluorescence quenching measurements, nephelometry, and dynamic light scattering experiments, the astringency-masking effect of polysaccharides has been essentially ascribed to two candidate molecular mechanisms [47, 48]: either soluble ternary protein–polyphenol–polysaccharide complexes are formed or polysaccharides bind to the polyphenol, which is then no longer available for salivary protein interactions [47, 49]. To the best of our knowledge, none of these postulated theories has yet been experimentally verified for native saliva-polyphenol mixtures.

**Fig 5:**
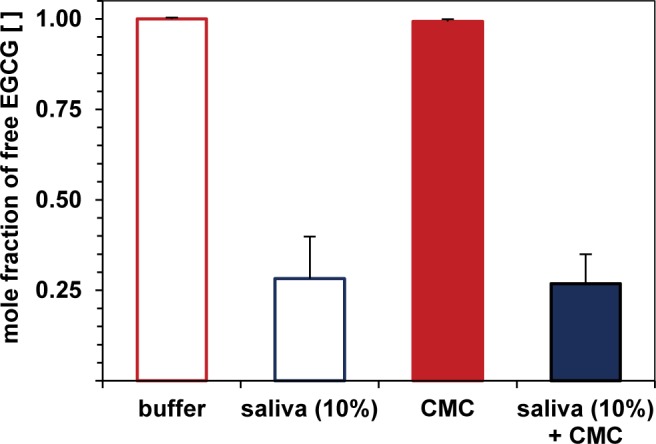
Unbound (-)-epigallocatechin gallate (EGCG) in pure buffer (reference), 10% (v/v) human whole saliva, 1% (w/v) carboxymethyl cellulose (CMC), and a combination of both. Unbound EGCG was quantified by single qHNMR measurements and is expressed as mole fractions. Error bars denote the standard deviation as obtained from integrating all the distinct, non-overlaid proton signals of EGCG. Data points behind means are provided in S3 Table.

To investigate the effect of CMC on polyphenol–protein binding, qHNMR studies were performed on EGCG incubated in the presence of 10% (v/v) human saliva, 1% (w/v) CMC, and a mixture of 10% saliva and 1% CMC. As expected, the presence of 10% saliva reduced the free mole fraction of EGCG by approximately 70% (Fig 5). In comparison, CMC did not affect the ^1^H-NMR signals of EGCG, and a binary CMC-saliva mixture spiked with an equal amount of EGCG revealed the same reduced amount of unbound EGCG as saliva diluted to 10%. As the presence of 1% CMC, which significantly masked the perceived astringency of EGCG, did not disrupt the salivary protein–EGCG complexes, an inhibitory effect on the binding interaction associated with a competition mechanism seems rather unlikely. Alternatively, EGCG may still interact with protein when embedded in a CMC coating in the form of ternary complexes as proposed for mixtures of α-amylase with procyanidins and pectins [47, 50]. Consequently, the enhanced solubility of carbohydrate-covered polyphenol–protein complexes in aqueous media, in addition to the inherent lubricating effect of CMC [51], may oppose the development of a roughening oral sensation *in vivo*.

## Conclusions

The label-free qHNMR titration approach was revealed to be a versatile tool to compare and classify the binding between low-affinity ligands and proteins in solution. The method essentially makes use of ^1^H-NMR signal attenuation of a low molecular weight ligand due to protein interactions and accurate quantitation of the mole fraction of a non-bound ligand at varied protein concentrations. Ligand-focused titration was applied to determine the binding affinities of selected polyphenols with salivary mucin. Particularly, the galloylated flavan-3-ols ECG and EGCG revealed a pronounced protein binding capacity. NMR spectroscopy unveiled the presence of exclusively soluble polyphenol–protein complexes for EC, EGC, methyl gallate, and rutin. This study shows that polyphenol-induced astringency may not require protein precipitation. Whereas soluble complex formation with lubricating salivary mucins may explain the mouth-puckering, astringent effect, particularly of low molecular weight catechins, the reported velvety astringency of flavonol glycosides is more likely to be due to interaction with other salivary proteins. The identity of these proteins will need to be investigated in future studies.

Ligands were further ranked with respect to their affinity for human whole saliva by single comparative NMR measurements. Single-point measurements agreed very well with the NMR titrations and enabled classification of the binding strength of the ligands. This approach was revealed to be a time-saving alternative, in case a crude ranking of ligands according to their relative binding affinities is sought within an experimental trial. As the protein affinity values were successfully linked to the level of astringency of these polyphenols, the NMR-based method may be useful to prospectively predict oral astringency *in vitro*, at least that of polyphenols. As the “anti-astringent” carboxymethyl cellulose was not found to affect protein–polyphenol interactions in a competitive qHNMR experiment, the formation of ternary protein–polyphenol–polysaccharide complexes may explain the astringency-masking effect of this polysaccharide. The method described in this paper has the potential to be of broad interest beyond the food science community. It may, for example, allow for relatively quantifying the affinity of small molecules with other biological specimens, such as blood plasma, tissue extract, whole cell lysate, or cell culture medium. Furthermore, the method may be suited to advance the understanding of intermolecular interactions under physiologically relevant *in vitro* conditions that mimic the cellular milieu [52]. Based on the data shown here for polyphenols, single-point quantitative NMR measurements also appear to be a promising approach in the discovery of bioactives, where the binding affinities of a multitude of drug candidates need to be rapidly tested in solution.

## Supporting information

S1 Table Data points behind means (Fig 3). (PDF)

S2 Table Data points behind means (Fig 4). (PDF)

S3 Table Data points behind means (Fig 5). (PDF)
