# Supplementary material for: Label-free quantitative 1H NMR spectroscopy to study low-affinity ligand–protein interactions in solution: A contribution to the mechanism of polyphenol-mediated astringency
Source: PLoS One. 2017 Sep 8;12(9):e0184487. doi: 10.1371/journal.pone.0184487 (PMC5590944; doi:10.1371/journal.pone.0184487)
Supplement: S1 Table — (PDF) [file pone.0184487.s001.pdf]

# Supporting Information (S1 Table)

Data points behind means (Fig 3).

# Label-free quantitative $^{1}$H NMR spectroscopy to study low-affinity ligand–protein interactions in solution: A contribution to the mechanism of polyphenol-mediated astringency

*Judith Delius, Oliver Frank, and Thomas Hofmann*[*]

[*]E-mail: thomas.hofmann@tum.de

| EC | 7.07 ppm | 7.00 ppm | 6.15 ppm | 6.11 ppm |
|---|---|---|---|---|
| mucin [µmol/L] | EC [µmol/L] | | | |
| 0 | 2.50 | 2.51 | 2.52 | 2.54 |
| 0.175 | 2.31 | 2.41 | 2.34 | 2.35 |
| 0.250 | 2.28 | 2.36 | 2.22 | 2.17 |
| 0.625 | 2.00 | 2.09 | 1.64 | 1.66 |
| 0.875 | 1.90 | 1.96 | 1.38 | 1.40 |
| 1.250 | 1.76 | 1.86 | 1.24 | 1.25 |
| 1.875 | 1.46 | 1.49 | 0.84 | 0.86 |
| 2.500 | 1.30 | 1.32 | 0.71 | 0.74 |

| ECG | 7.03 ppm | 6.90 ppm | 6.19 ppm |
|---|---|---|---|
| mucin [µmol/L] | ECG [µmol/L] | | |
| 0 | 2.53 | 2.55 | 2.50 |
| 0.063 | 2.32 | 2.22 | 2.21 |
| 0.125 | 2.07 | 2.14 | 1.78 |
| 0.150 | 1.77 | 1.86 | 1.56 |
| 0.188 | 1.59 | 1.70 | 1.37 |
| 0.250 | 1.33 | 1.47 | 1.13 |
| 0.375 | 0.91 | 1.06 | 0.77 |
| 0.500 | 0.75 | 0.89 | 0.58 |
| 1.000 | 0.33 | 0.39 | 0.29 |
| 1.250 | 0.27 | 0.36 | 0.22 |

| EGC | 6.69 ppm | 6.18 ppm | 6.14 ppm |
|---|---|---|---|
| mucin [µmol/L] | EGC [µmol/L] | | |
| 0 | 2.50 | 2.50 | 2.50 |
| 0.375 | 2.52 | 2.32 | 2.32 |
| 1.250 | 2.03 | 1.16 | 1.24 |
| 1.875 | 1.34 | 0.74 | 0.78 |
| 2.500 | 1.05 | 0.58 | 0.62 |

| EGCG | 7.04 ppm | 6.66 ppm | 6.20 ppm |
|---|---|---|---|
| mucin [µmol/L] | EGCG [µmol/L] | | |
| 0 | 2.49 | 2.50 | 2.50 |
| 0.125 | 1.94 | 1.97 | 1.65 |
| 0.188 | 1.51 | 1.61 | 1.24 |
| 0.250 | 1.18 | 1.32 | 0.94 |
| 0.375 | 0.78 | 0.98 | 0.69 |
| 0.500 | 0.57 | 0.78 | 0.52 |
| 0.625 | 0.52 | 0.65 | 0.42 |
| 0.750 | 0.44 | 0.57 | 0.36 |
| 0.875 | 0.37 | 0.48 | 0.28 |
| 1.000 | 0.32 | 0.43 | 0.26 |
| 1.250 | 0.19 | 0.3 | 0.17 |

| methyl gallate | measurement 1 | measurement 2 |
|---|---|---|
| | 7.17 ppm | |
| mucin [µmol/L] | methyl gallate [µmol/L] | |
| 0 | 2.50 | 2.50 |
| 0.125 | 2.18 | - |
| 0.375 | 2.06 | - |
| 0.625 | 1.99 | 1.94 |
| 0.875 | 1.84 | - |
| 1.250 | 1.72 | 1.77 |
| 1.875 | 1.53 | - |
| 2.500 | 1.40 | 1.44 |

| rutin | 7.43 ppm | 6.88 ppm | 6.16 ppm |
|---|---|---|---|
| mucin [µmol/L] | rutin [µmol/L] | | |
| 0 | 2.50 | 2.50 | 2.51 |
| 1.000 | 2.23 | 2.49 | 2.12 |
| 2.500 | 1.60 | 2.25 | 1.38 |
| 5.000 | 1.00 | 1.77 | 0.68 |
