# Supplementary material for: Label-free quantitative 1H NMR spectroscopy to study low-affinity ligand–protein interactions in solution: A contribution to the mechanism of polyphenol-mediated astringency
Source: PLoS One. 2017 Sep 8;12(9):e0184487. doi: 10.1371/journal.pone.0184487 (PMC5590944; doi:10.1371/journal.pone.0184487)
Supplement: S2 Table — (PDF) [file pone.0184487.s002.pdf]

Supporting Information (S2 Table)

Data points behind means (Fig 4).

Label-free quantitative ^{1}H NMR spectroscopy to study low-affinity ligand–protein interactions in solution: A contribution to the mechanism of polyphenol-mediated astringency

*Judith Delius, Oliver Frank, and Thomas Hofmann**

*E-mail: thomas.hofmann@tum.de

EC	7.07 ppm	7.00 ppm	6.15 ppm	6.11 ppm
saliva	EC [µmol/L]			
1	2.37	2.40	2.37	2.22
2	2.04	2.07	1.64	1.65

ECG	7.03 ppm	6.90 ppm	6.19 ppm
saliva	ECG [µmol/L]		
1	0.00	0.00	0.00
2	0.00	0.00	0.00

EGC	6.69 ppm	6.18 ppm	6.14 ppm
saliva	EGC [µmol/L]		
1	2.16	2.10	2.00
2	1.95	1.50	1.48

EGCG	7.04 ppm	6.66 ppm	6.20 ppm
saliva	EGCG [µmol/L]		
1	0.00	0.00	0.00
2	0.00	0.00	0.00

methyl gallate	7.17 ppm
saliva	methyl gallate [µmol/L]
1	2.42
2	2.32

rutin	7.43 ppm	6.88 ppm	6.16 ppm
saliva	rutin [µmol/L]		
1	2.12	2.39	2.39
2	2.35	2.49	2.40
