# Supplementary material for: Label-free quantitative 1H NMR spectroscopy to study low-affinity ligand–protein interactions in solution: A contribution to the mechanism of polyphenol-mediated astringency
Source: PLoS One. 2017 Sep 8;12(9):e0184487. doi: 10.1371/journal.pone.0184487 (PMC5590944; doi:10.1371/journal.pone.0184487)
Supplement: S3 Table — (PDF) [file pone.0184487.s003.pdf]

Supporting Information (S3 Table)

Data points behind means (Fig 5).

Label-free quantitative ^{1}H NMR spectroscopy to study low-affinity ligand–protein interactions in solution: A contribution to the mechanism of polyphenol-mediated astringency

Judith Delius, Oliver Frank, and Thomas Hofmann[*]

[*]E-mail: thomas.hofmann@tum.de (TH)

EGCG in	7.04 ppm	6.66 ppm	6.20 ppm
	EGCG [µmol/L]		
buffer	2.49	2.5	2.51
saliva (10%)	0.81	0.93	0.38
CMC	2.47	2.48	2.50
saliva (10%) + CMC	0.74	0.83	0.44
